# TSPAN9 suppresses the chemosensitivity of gastric cancer to 5-fluorouracil by promoting autophagy

**DOI:** 10.1186/s12935-019-1089-2

**Published:** 2020-01-03

**Authors:** Yaoyue Qi, Weiwei Qi, Shihai Liu, Libin Sun, Aiping Ding, Guohong Yu, Hui Li, Yixuan Wang, Wensheng Qiu, Jing Lv

**Affiliations:** 1 Qingdao University, Qingdao, Shandong China; 2 Department of Oncology, Affiliated Hospital of Qingdao University, Qingdao, Shandong China; 3 Key Laboratory of Cancer Molecular and Translational Research, Qingdao University, Qingdao, Shandong China; 4 Central Laboratory, Affiliated Hospital of Qingdao University, Qingdao, Shandong China

**Keywords:** TSPAN9, Gastric cancer, Autophagy, Chemoresistance

## Abstract

**Background:**

The issue of drug resistance in gastric cancer has attracted global attention. TSPAN9, a 4-transmembrane protein that plays an important role in tumor progression and signal transduction, has been found to be closely related to tumor invasion, metastasis, and autophagy.

**Methods:**

Immunoblotting was used to evaluate TSPAN9 expression in parental and drug-resistant gastric cancer cells. Functional assays, such as the CCK-8 assay, were used to detect the proliferation of gastric cancer cells and the response of TSPAN9 to 5-fluorouracil (5-FU). Western blotting was used to analyze the expression of constituents of the PI3K/AKT/mTOR-mediated autophagy pathway induced by TSPAN9. Coimmunoprecipitation was performed to assess the specific mechanism by which TSPAN9 affects the PI3K pathway.

**Results:**

We demonstrated that TSPAN9 is overexpressed in 5-FU-resistant cells compared to parental cells. 5-FU-mediated inhibition of cell proliferation can be significantly restored by increasing TSPAN9 expression, and inhibiting this expression in drug-resistant cells can restore the sensitivity of the cells to 5-FU. In addition, TSPAN9 also significantly promoted autophagy in gastric cancer cells in vitro. Further studies indicated that TSPAN9 downregulates the expression of PI3K and proteins associated with PI3K-mediated autophagy. In addition, TSPAN9 interacts with PI3K and inhibits its catalytic activity.

**Conclusion:**

The current study reveals the important role of TSPAN9 in drug resistance to 5-FU in gastric cancer. It also provides a new target to clinically address drug-resistant gastric cancer and will contribute to the treatment strategy of this disease.

## Background

Gastric cancer is one of the most common malignant tumors in the world; in China, newly diagnosed gastric cancer cases account for more than 40% of worldwide cases each year, which corresponds to a high incidence [1, 2]. Because early symptoms are not obvious, patients are often in advanced stages at the time of diagnosis; thus, chemotherapy is the main treatment for these patients [3, 4]. 5-Fluorouracil (5-FU) is the cornerstone of gastric cancer chemotherapy and functions by blocking DNA production in tumor cells via inhibition of thymidylate synthase activity [5, 6]. However, problems relating to 5-FU drug resistance have become a major obstacle to treating gastric cancer [7]. Therefore, there is an urgent need to elucidate the important molecular mechanisms of 5-FU drug resistance, which will help improve the efficacy of chemotherapy and the prognosis of patients.

Autophagy, one of the important physiological processes of cells, involves the formation of autophagosomes through the bilayer membrane that are to be degraded by lysosomes in order to meet the metabolic needs of the cells themselves and recycle the organelles [8, 9]. Autophagy is closely related to cell differentiation and apoptosis as well as the occurrence and development of various diseases [10]. In the advanced stages of tumor development, the induction of autophagy allows cancer cells to survive under low nutrient and hypoxic conditions [11]. Chemotherapy drugs have been reported to induce autophagy by blocking the apoptotic pathway to protect tumor cells from cytotoxic death [12]. However, autophagy plays an important role in the development of chemotherapy resistance during the initiation and progression of gastric cancer.

Tetraspanins, also known as tetraspans, TSPANs, or the transmembrane 4 superfamily (TM4SF), are a large family of evolutionarily conserved four-transmembrane-domain proteins [13]. Structurally, TSPANs consist of four transmembrane segments, a small extracellular region and a large extracellular loop (LEL) [14]. The homology among the family members is highly conserved except for the small variable domains located within the LEL, which may result in differences in function between isoforms [15]. In previous studies, TSPAN9 was shown to inhibit the proliferation and migration of gastric cancer cells by enhancing autophagy [16]. Currently, autophagy is one of the key mechanisms related to drug resistance, so we suspected that TSPAN9 is involved in this resistance. Furthermore, we analyzed TSPAN9 expression in gastric cancer cells and 5-FU-resistant gastric cancer cells and found that it was high in drug-resistant cells, which led us to further explore this phenomenon. In the present study, we demonstrate that TSPAN9 blocks PI3K–Akt–mTOR signaling by interacting with PI3K, which enhances autophagy and leads to 5-FU resistance in gastric cancer cells. Our research suggests that TSPAN9 may be a novel mechanism for inducing drug resistance in cancer cells.

## Methods

### Cell culture

AGS and MGC803 cell lines were obtained from the Shanghai Institutes for Biological Sciences (Shanghai, People’s Republic of China) and were grown in RPMI 1640 (Gibco, CA, USA) containing 10% fetal bovine serum (FBS; Thermo Fisher Scientific, MA, USA) and 1% penicillin/streptomycin (HyClone, UT, USA) under standard growth conditions (Table 1).

**Table 1:** Resistance Index of the parental gastric cancer lines and their 5-fluorouracil resistant cell lines to 5-FU

Cell line	IC50 of 5-FU (μM)		Resistance Index (RI)
	Parent cell	Resistance cell	
AGS	18.52 ± 0.83	124.90 ± 0.87	6.74
MGC803	14.93 ± 0.92	98.40 ± 0.77	6.59

### Establishment of 5-fluorouracil-resistant cells

AGS/R and MGC803/R cells are resistant to 5-fluorouracil (5-FU); they were established by gradually exposing the parental AGS and MGC803 cell lines to increasing concentrations of 5-FU, ranging from 0.1 to 100 µg/ml over at least a 6-month period.

### Cell viability assay

The viability of cells was measured using a Cell Counting Kit-8 assay kit (Sigma-Aldrich, Shanghai, China). Cells (1 × 10^3^ cells/well) were seeded in 96-well plates, grown overnight at 37 °C and then treated with varying concentrations of 5-FU for the indicated times. At the end of treatment, 10 µl of CCK-8 solution was added and incubated for 4 h. The absorbance at 450 nm was measured on a microplate reader (Model 680 microplate reader, Bio-Rad Laboratories). The values are presented as the mean ± SD for triplicate wells and normalized to those of the control group to determine the % viability.

### Western blot analysis

Primary antibodies targeting the following proteins were used: TSPAN9 (1:1000, Abcam), LC3 (1:1000, CST), P62 (1:1000, CST), Beclin1 (1:1000, Bioworld), PI3K (1:1000, CST), p-PI3K (1:1000, CST), AKT (1:1000, CST), p-AKT (1:1000, CST), mTOR (1:1000, CST), and p-mTOR (1:1000, CST). Cells were collected and lysed in ice-cold RIPA lysis buffer (1× Tris-buffered saline, 1% Nonidet P-40, 0.5% sodium deoxycholate, and 0.1% sodium dodecyl sulfate (SDS). Then, 40 µg of cell lysate from each sample was separated by SDS-polyacrylamide gel electrophoresis (PAGE) through a 10% gel. Electrophoretic transfer of the proteins from the gels to nitrocellulose membranes was carried out in a transblotting chamber. After the membranes were blocked with 5% nonfat milk (w/v) in phosphate-buffered saline (PBS) for 1 h to inhibit nonspecific binding, they were incubated with primary antibodies at room temperature for 2 h. The membranes were then washed 3 times with PBST before secondary antibodies diluted in PBST were incubated with the membranes for 40 min at room temperature. The following secondary antibodies were used: anti-rabbit IgG HRP-linked antibody and anti-mouse IgG HRP-linked antibody (Sigma, 1:6000). The membranes were washed 6 times with PBST for 1 h, and the protein bands were visualized by chemiluminescence (Clarity Western ECL; Bio-Rad).

### RT-PCR

TSPAN9 expression levels were measured by qPCR analysis using cDNA synthesized from 5 µg of total RNA and a reverse transcription kit (Promega, Madison, WI). In all, 1 µl of cDNA was used for PCR, and duplicate reactions were performed for each sample using ABI Power SYBR Green PCR Master Mix (Applied Biosystems, Warrington) with gene-specific primers on an ABI StepOnePlus instrument (Applied Biosystems). The RNA levels were normalized to those of GAPDH, and gene expression was quantified according to the 2−^Δ^Ct method.

### Transmission electron microscopy

Following trypsinization, cells were fixed in 2.5% glutaraldehyde and 0.1 M cacodylate buffer (pH 7.4) for 2 h and then rinsed twice with precooled PBS. After they were washed with buffer solution, the cells were postfixed in 1% osmium tetroxide (OsO4) and 0.1 M cacodylate buffer (pH 7.4), washed with buffer solution, dehydrated through a graded series of ethanol, and embedded in epoxy resin. The ultrastructures of cells undergoing autophagy were observed and imaged via transmission electron microscopy (TEM) (VEGA TESCAN, Czech Republic).

### Coimmunoprecipitation

Antibodies against TSPAN9 (1:1000, Abcam) and p55 (1:1000, CST) were used. First, Pierce Protein A Agarose beads (Thermo Scientific, IL, USA) were washed in a solution containing 1% BSA and 10% SDS in PBS, followed by four washes with PBS. Beads were resuspended in PBS containing 1% BSA, to which 4 μl of the indicated antibody was added and incubated for 4 h at 4 °C. The beads were then washed 4 times in PBS to remove unbound antibodies prior to immunoprecipitation. Following the immunoprecipitation reaction, samples were boiled for 3 min in 2× SDS loading buffer to elute bound proteins, which were separated by SDS-PAGE. After silver staining of the gels, mass spectrometry was used to identify the coimmunoprecipitated proteins.

### GFP-LC3 puncta assay

Cells were seeded on 42-mm glass cover slips at a density of 1 × 10^5^ cells/ml, infected with recombinant plasmid expressing GFP-LC3 proteins at a multiplicity of infection (MOI) of 100, and then incubated at the indicated conditions for 24 h. GFP-LC3 puncta were visualized under a confocal microscope (FV-1000; Olympus) and analyzed. The numbers of GFP-LC3 puncta in 6 randomly selected high-power fields were assessed; puncta from at least 20 cells were counted per sample, and each experiment was carried out 3 times.

### Statistical analysis

All analyses were performed using GraphPad Prism Version 6.0 (GraphPad Software Inc., San Diego, CA). Data are presented as the means ± SDs of triplicate samples or at least three independent experiments. All statistically significant data were indicated by p < 0.05. Statistical comparisons between the control and treatment groups were determined using unpaired Student’s t test or one-way ANOVA.

## Results

### TSPAN9 is overexpressed in 5-FU-resistant gastric cancer cells

**Fig. 1:**
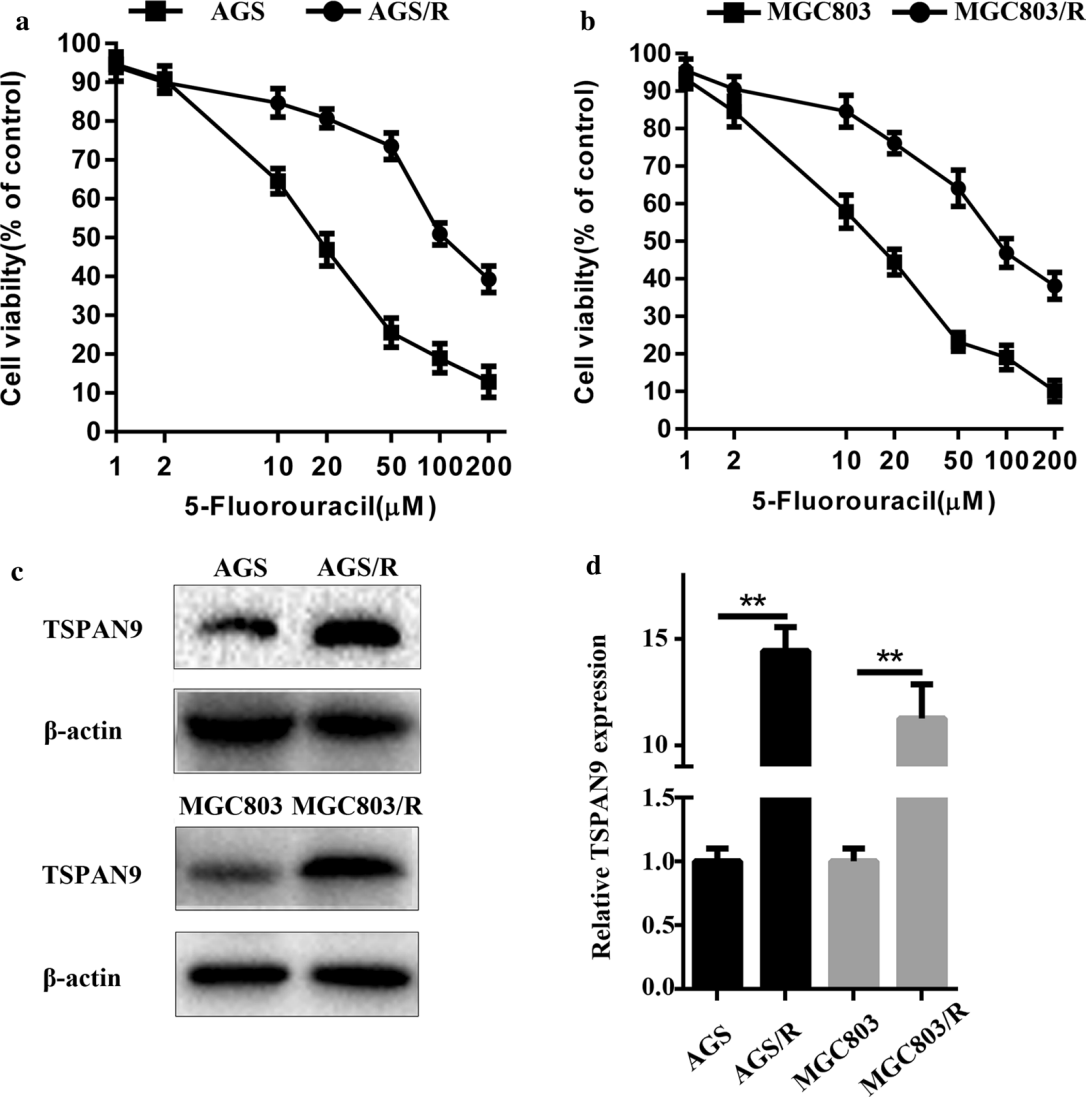
TSPAN9 is overexpressed in 5-FU-resistant gastric cancer cells. All cell lines were cultured with various concentrations (1, 2, 10, 20, 50, 100 and 200 µM) of 5-FU for 72 h. Cell proliferation activity was measured by the CCK-8 assay. Each data point represents the mean ± SD (n = 6). All 5-FU-resistant cell lines were more resistant to 5-FU than their respective parental cell lines. **a** The sensitivity of the parental gastric cancer cell line AGS and its 5-FU-resistant cell line (AGS/R). **b** Cell proliferation activity of the parental gastric cancer cell line MGC803 and its 5-FU-resistant cell line (MGC803/R). **c** Western blot assay of TSPAN9 in parental and drug-resistant gastric cancer cell line cells, with β-actin serving as a loading control. **d** qRT-PCR was employed to assess TSPAN9 expression in parental and drug-resistant gastric cancer cell lines (n = 3). GAPDH served as an internal reference. *p < 0.05, **p < 0.01; Student’s t test

To investigate the molecular mechanism of 5-FU resistance in gastric cancer cells, we constructed 5-FU-resistant gastric cancer cell lines, AGS/R and MGC803/R, by gradually exposing parental AGS and MGC803 cells to increasing concentrations of 5-FU for 6 months. Thereafter, the proliferative potential of the parental and drug-resistant cells was analyzed by the CCK-8 assay. As shown in Fig. 1, the parental and drug-resistant cells were treated with different concentrations of 5-FU for 72 h before cell viability was measured, and the results showed that the AGS and MGC803 parental cells were significantly more sensitive to 5-FU than were the AGS/R and MGC803/R cells. Therefore, the AGS/R and MGC803/R cell lines exhibited 5-FU resistance. Next, we analyzed TSPAN9 expression in the parental and drug-resistant cells using immunoblotting and real-time PCR, which confirmed that AGS/R and MGC803/R cells had much higher levels of TSPAN9 expression than did the respective parental cells (Fig. 1c, d).

### TSPAN9 can reduce the sensitivity of gastric cancer cells to 5-FU

**Fig. 2:**
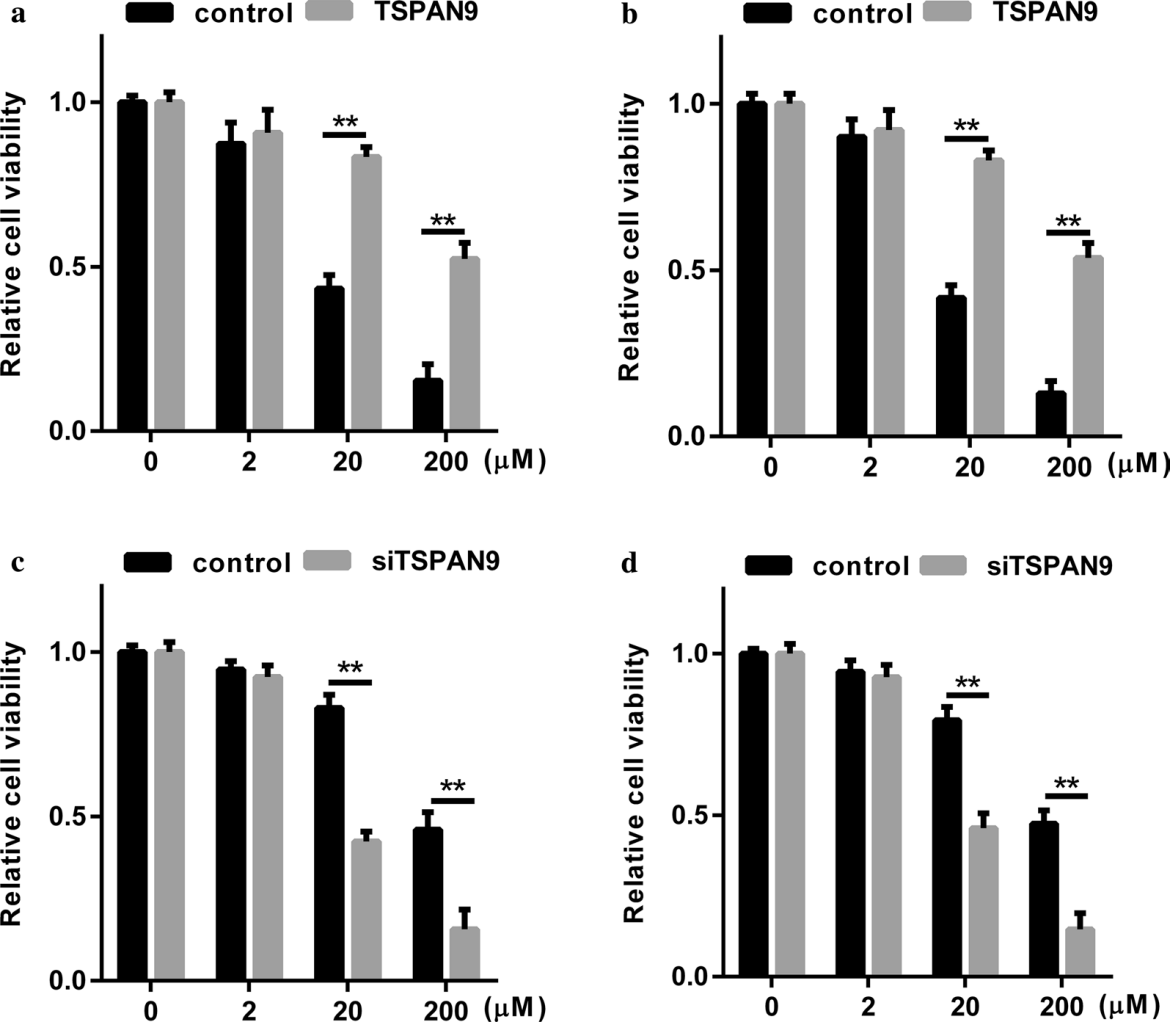
TSPAN9 can reduce the sensitivity of gastric cancer cells to 5-FU. **a** AGS cells transfected with a TSPAN9-overexpressing plasmid or empty vector plasmid were treated with 0, 2, 20, or 200 µM 5-FU for 48 h. Cell viability was detected by the MTT assay. **b** MGC803 cells transfected with TSPAN9-overexpressing plasmid or empty vector plasmid were treated with 0, 2, 20, or 200 µM 5-FU for 48 h. Cell viability was detected by the MTT assay. **c** AGS/R cells transfected with siTSPAN9 or empty vector plasmid were treated with 0, 2, 20, or 200 µM 5-FU for 48 h. Cell viability was detected by the MTT assay. **d** MGC803/R cells transfected with siTSPAN9 or empty vector plasmid were treated with 0, 2, 20, or 200 µM 5-FU for 48 h. Cell viability was detected by the MTT assay. *p < 0.05, **p < 0.01; Student’s t test

To investigate whether the level of TSPAN9 expression in cells is related to 5-FU resistance, we transfected AGS and MGC803 cells with TSPAN9 overexpression plasmids and then treated the cells with 5-FU. The CCK-8 assay showed that compared to the control cells, cells with TSPAN9 overexpression showed significantly reduced 5-FU-induced cell death and increased 5-FU resistance (Fig. 2a, b). Afterwards, we knocked down TSPAN9 in AGS/R and MGC803/R cells using TSPAN9-shRNA plasmids and found that 5-FU resistance was significantly reduced (Fig. 2c, d). These data suggest that high levels of TSPAN9 may protect AGS and MGC803 cells from 5-FU-driven cytotoxic damage, while knocking down TSPAN9 in cells may reverse this resistance.

### TSPAN9 increases the level of autophagy in gastric cancer cells

**Fig. 3:**
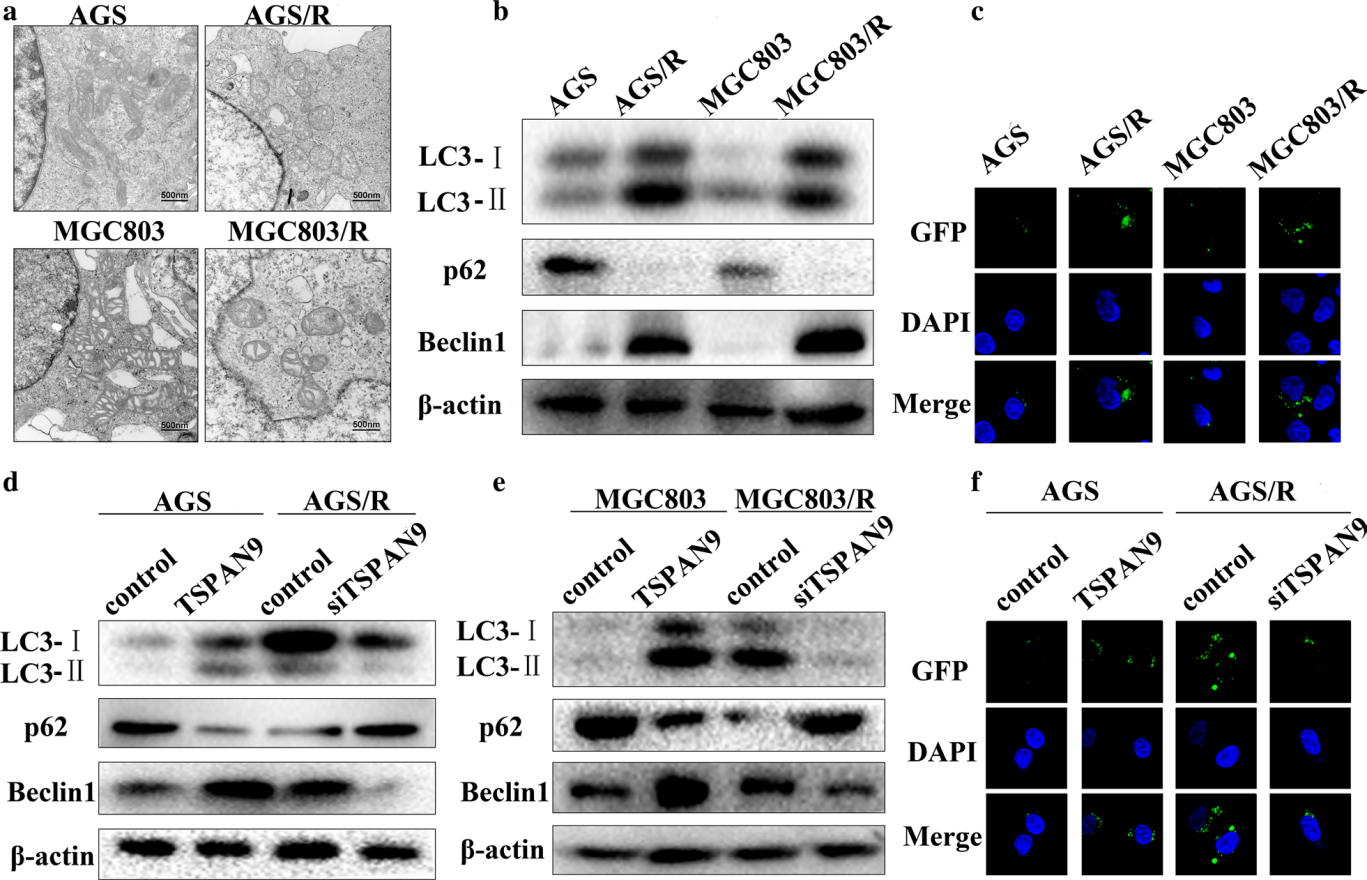
TSPAN9 increases the level of autophagy in gastric cancer cells. **a** AGS, AGS/R, MGC803 and MGC803/R cells were treated with 100 µM 5-FU for 48 h and then subjected to TEM. Autophagosomes are indicated by red arrows. **b** AGS, AGS/R, MGC803 and MGC803/R cells were treated with 100 µM 5-FU for 48 h followed by immunoblotting with the indicated antibodies. **c** AGS, AGS/R, MGC803 and MGC803/R cells transduced with GFP-LC3 plasmid were treated with 100 µM 5-FU for 24 h. After the cells were stained with DAPI, the fluorescence of the cells was detected under a confocal microscope. **d** Western blotting was used to assess the levels of autophagy-associated proteins after the transfection of TSPAN9 in AGS cells and TSPAN9-shRNA in AGS/R cells. **e** Western blotting was used to assess the levels of autophagy-associated proteins after the transfection of TSPAN9 in MGC803 cells and TSPAN9-shRNA in MGC803/R cells. **f** After transfecting TSPAN9 into AGS cells and TSPAN9-shRNA in AGS/R cells infected with GFP-LC3 plasmid, the cells were treated with 100 µM 5-FU for 24 h. The cells were then stained with DAPI, and the fluorescence was detected under a confocal microscope

To understand the mechanisms involved in the resistance of human gastric cancer cells to 5-FU, we first observed the morphology of the parental and drug-resistant cells. TEM showed that upon exposure to 5-FU, the drug-resistant cells had higher levels of autophagy than did the parental cells (Fig. 3a). Since autophagy has been found to be involved in cancer cell resistance [17, 18], we investigated whether TSPAN9 enhances autophagy levels. The Western blot results showed that after 5-FU treatment, the drug-resistant cells had a higher LC3-II/I ratio and lower SQSTM1/p62 protein levels than did the parental cells (Fig. 3b). To compare the actual autophagy flux in the parental and drug-resistant cells, we infected cells with a recombinant plasmid expressing a GFP-LC3 fusion protein (GFP-LC3). The results showed that after 5-FU stimulation, the drug-resistant cells had more yellow spots than did the parental cells (Fig. 2c). These results indicate that AGS/R and MGC803/R cells have higher autophagy levels than their respective parental cells. We then knocked down TSPAN9 in AGS/R and MGC803/R cells by transfecting the cells with siTSPAN9. As shown in Fig. 3, the LC3-II/I ratio was significantly reduced, and the level of SQSTM1/p62 was significantly increased in drug-resistant cells with TSPAN9 knockdown. In addition, confocal microscopy images showed a decrease in the number of siTSPAN9A-induced autophagy flux spots. As expected, TSPAN9 overexpression in AGS and MGC803 cells increased the LC3-II/I ratio and decreased SQSTM1/p62 levels (Fig. 3d, e). The above experiments demonstrated that the autophagy activity in drug-resistant cells is increased and that TSPAN9 promotes autophagy.

### TSPAN9 affects the sensitivity of gastric cancer cells to 5-FU by autophagy

**Fig. 4:**
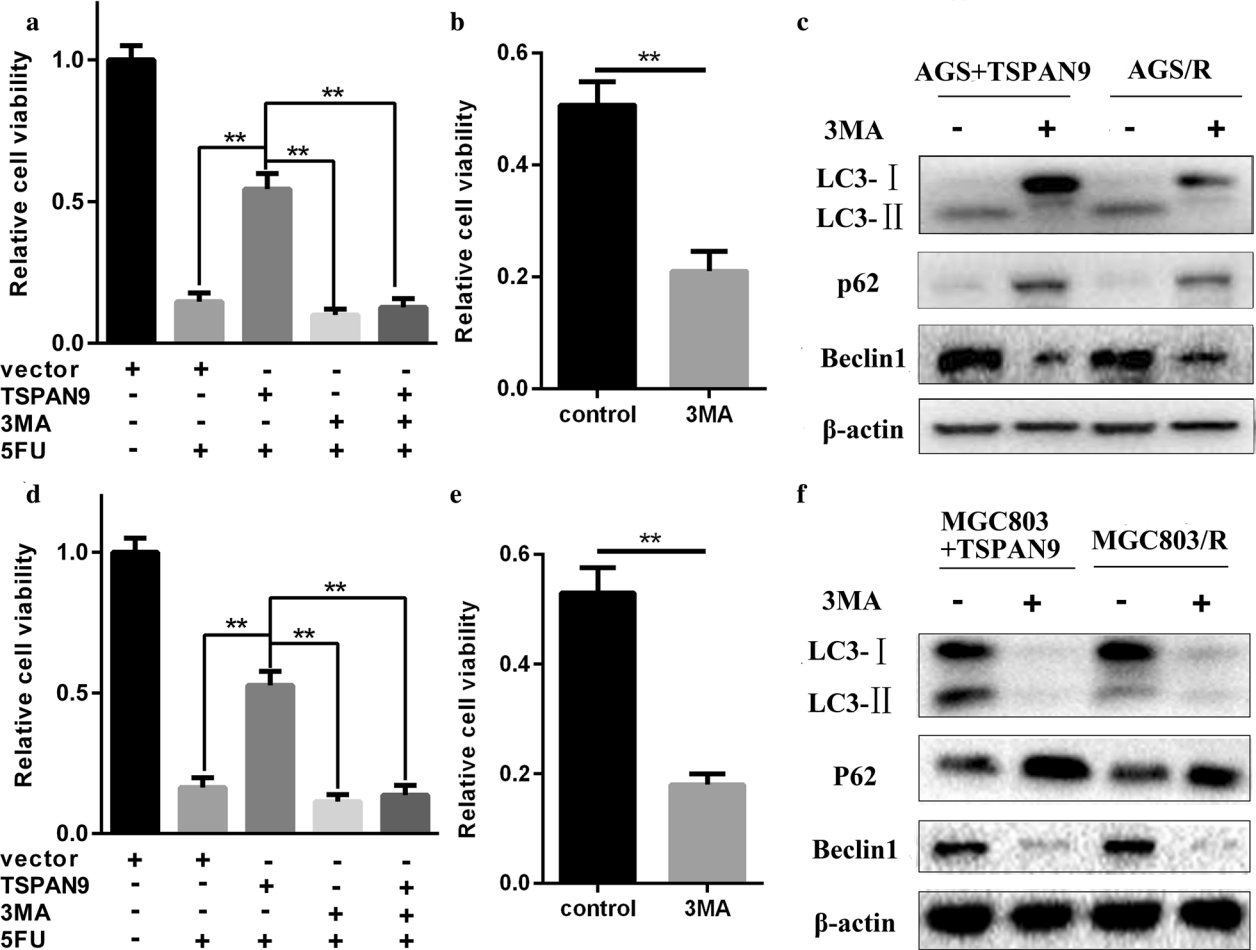
TSPAN9 affects the sensitivity of gastric cancer cells to 5-FU by mediating autophagy. **a** AGS cells were treated with 3-MA (a kind of autophagy inhibitor) and transfected with TSPAN9 plasmids prior to the addition of 100 µM 5-FU for 24 h. Cell viability was assessed by the CCK-8 assay. **b** AGS/R cells were treated with 3-MA and transfected with 100 µM 5-FU for 24 h. Cell viability was assessed by the CCK-8 assay. **c** AGS cells transfected with a TSPAN9-overexpressing vector and AGS/R cells treated with 3-MA were subjected to immunoblotting with the indicated antibodies. **d** MGC803 cells were treated with 3-MA and TSPAN9 plasmids and then treated with 100 µM 5-FU for 24 h. Cell viability was assessed by the CCK-8 assay. **e** MGC803/R cells were transfected with 3-MA and then treated with 100 µM 5-FU for 24 h. Cell viability was assessed by the CCK-8 assay. **f** MGC803 cells transfected with a TSPAN9-overexpressing vector and MGC803/R cells treated with 3-MA were subjected to immunoblotting with the indicated antibodies. *p < 0.05, **p < 0.01; Student’s t test

These results indicate that TSPAN9 can promote autophagy and affect 5-FU resistance in gastric cancer cells. To assess whether TSPAN9 contributes to 5-FU resistance by inducing high levels of autophagy, we added the autophagy inhibitor 3-methyl adenine (3-MA) to resistant cells and to AGS and MGC803 cells transfected with the TSPAN9-overexpressing plasmid. As shown in Fig. 4, drug-resistant cells and cells overexpressing TSPAN9 treated with autophagy inhibitors showed significant inhibition in the TSPAN9-induced increase in autophagy and increased 5-FU-induced cell death. These results indicate that TSPAN9 protects gastric cancer cells from 5-FU-induced cell death by enhancing autophagy.

### TSPAN9 promotes autophagy through the PI3K/AKT/mTOR pathway

**Fig. 5:**
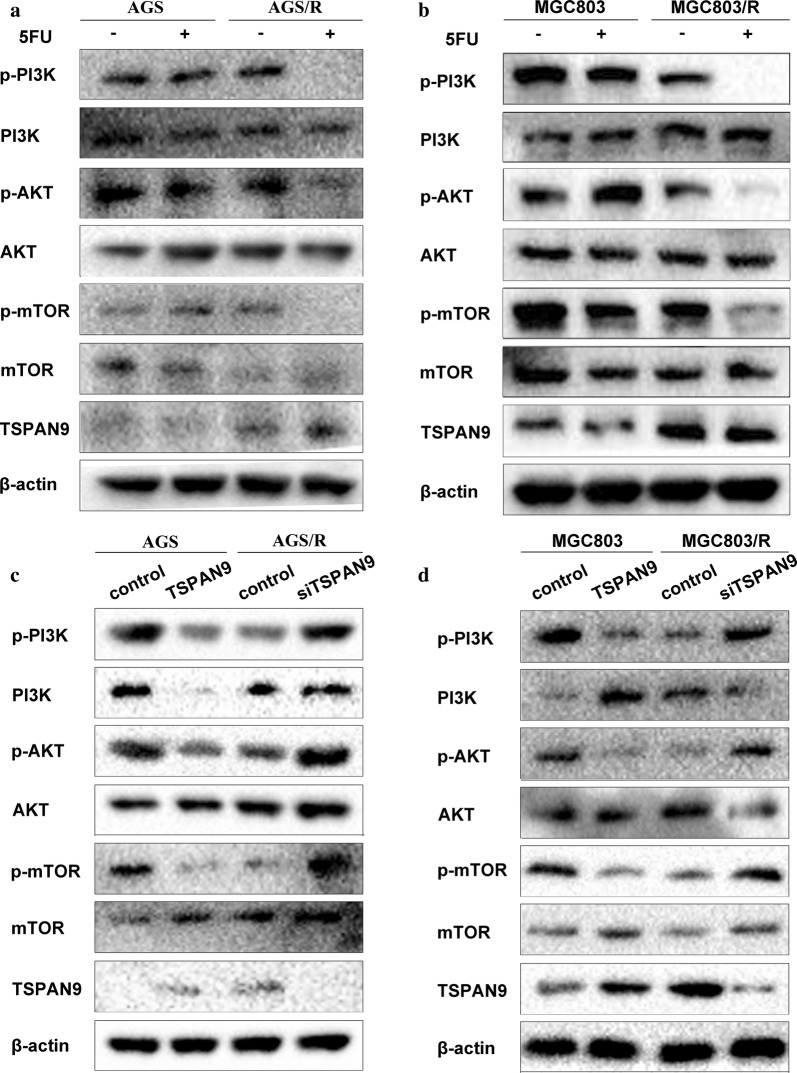
TSPAN9 promotes autophagy through the PI3K/AKT/mTOR pathway. **a**, **b** After AGS, AGS/R, MGC803 and MGC803/R cells were treated with 5-FU or vehicle (control) for 48 h, the levels of PI3K, AKT, and mTOR were assessed by Western blotting, with β-actin serving as a loading control. **b**, **c** AGS cells were transduced with siTSPAN9 and cultured in the presence or absence of a RAS inhibitor. **c**, **d** In AGS, AGS/R, MGC803 and MGC803/R cells transfected with TSPAN9 or a negative control (control) for 48 h, the levels of PI3K, AKT, and mTOR were assessed by Western blotting, with β-actin serving as a loading control

There are a variety of autophagy-related signaling pathways available in tumor cells, with the PI3K/AKT pathway as a classical activator of mTOR to induce autophagy [19, 20]. Upon analysis, we found that the levels of phosphorylated PI3K, Akt, and mTOR in the drug-resistant cells were lower than those in the parental cells, indicating that high levels of autophagy in the drug-resistant cells are associated with low levels of PI3K/AKT/mTOR activation (Fig. 5a, b). Further experiments showed that TSPAN9 overexpression in the parental cells significantly inhibited the activation of the PI3K–Akt–mTOR pathway. Conversely, TSPAN9 knockdown in the drug-resistant cells reversed the 5-FU-induced inhibition of PI3K–Akt–mTOR signaling (Fig. 5c, d). These results indicated that TSPAN9 promotes autophagy by inhibiting PI3K–Akt–mTOR pathway activation.

### TSPAN9 inhibits PI3K–Akt–mTOR signaling by binding to PIK3R3 (p55)

**Fig. 6:**
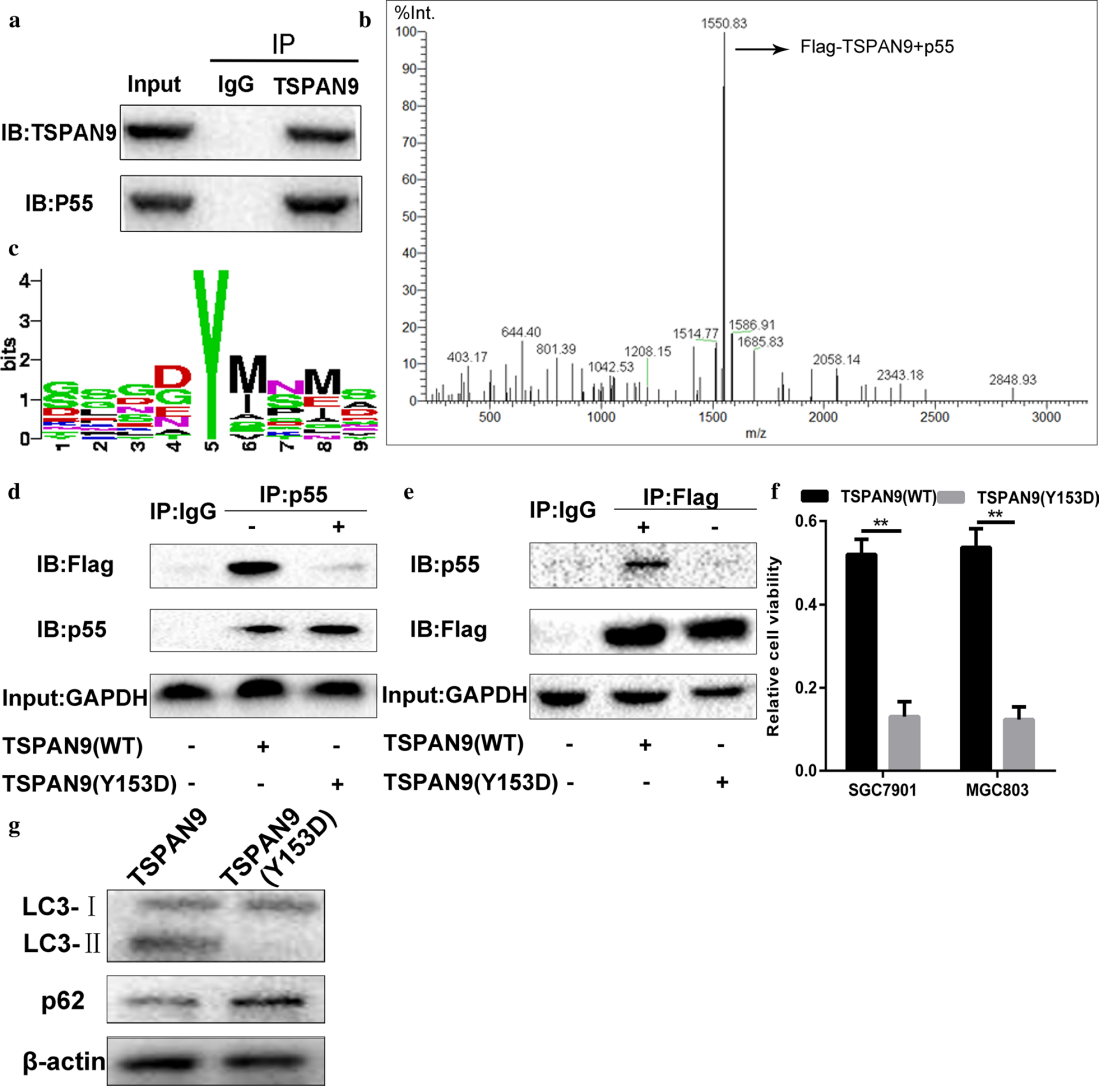
TSPAN9 inhibits PI3K–Akt–mTOR signaling by binding to PIK3R3 (p55). **a** Lysates from AGS/R cells stimulated with 5-FU were subjected to immunoprecipitation with anti-TSPAN9 followed by immunoblotting with anti-p55. **b** The endogenous TSPAN9 complex was analyzed by LC–MS–MS. **c** A schematic diagram of the TSPAN9 phosphorylation site. **d** AGS cells were transfected with TSPAN9 (WT) or TSPAN9 (Y153D). Lysates were subjected to immunoprecipitation with antibodies targeting p55 and TSPAN9. **e** AGS cells were transfected with either TSPAN9 (WT) or TSPAN9 (Y153D) plasmids and treated with 100 µM 5-FU for 24 h. Cell viability was assessed by the CCK-8 assay. **f** AGS cells were transfected with either TSPAN9 (WT) or TSPAN9 (Y153D) plasmids and subjected to immunoblotting for LC3 and P62

PIK3R3 (p55) is one of five regulatory PI3K subunits involved in the growth response of human cancers, and previous studies have shown that it can bind to TSPAN2 [21]. To elucidate the mechanism by which TSPAN9 regulates PI3K–Akt–mTOR signaling, we performed immunoprecipitation experiments to detect any specific association of endogenous TSPAN9 with PI3K in drug-resistant cells. The results showed that TSPAN9 was associated with PIK3R3 (p55) in drug-resistant cells stimulated with 5-FU (Fig. 6a). To confirm the interaction between TSPAN9 and p55, we performed LC–MS/MS detection. As expected, endogenous TSPAN9 in the drug-resistant cells was associated with p55 (Fig. 6b). The above results indicate that TSPAN9 specifically interacts with the p55 subunit of PI3K upstream of mTOR. Posttranslational modifications (PTMs), such as tyrosine phosphorylation, regulate protein–protein interactions (PPIs), which are critical for cell signal processing [22, 23]. Since the binding of TSPAN2 to PI3K is tyrosine phosphorylation-dependent, we treated the drug-resistant cells with a tyrosine kinase inhibitor (TKI) to analyze whether the binding of TSPAN9 to p55 is related to the phosphorylation status of TSPAN9. However, no increases in the autophagy levels were observed in the TKI-treated cells. Therefore, we speculated that the phosphorylation state of TSPAN9 plays a key role in its own function. Next, we predicted the phosphorylation site of TSPAN9 and found that the tyrosine at position 153 (Tyr153 or Y153) is a site with a high probability of phosphorylation. To verify whether phosphorylation is required for TSPAN9 to interact with p55, we constructed a TSPAN9-mutant plasmid in which tyrosine was replaced with asparagine (Y153D) and transfected this plasmids into gastric cancer cells. Immunoprecipitation and Western blotting experiments showed that compared to TSPAN9 (WT), TSPAN9 (Y153D) exhibited a significantly reduced interaction with p55 (Fig. 6d); thus, the phosphorylation of TSPAN9 is critical for the binding of TSPAN9 and p55. We then evaluated the importance of phosphorylation for TSPAN9 activity and found a significant decrease in the viability of 5-FU-treated cells transfected with TSPAN9 (Y153D) compared to that of 5-FU-treated cells transfected with TSPAN9 (WT). At the same time, immunoblotting experiments showed that overexpression of TSPAN9 (Y153D) did not increase autophagy levels. These results indicate that the phosphorylation of Tyr153 on TSPAN9 is important for the interaction of TSPAN9 with p55 and for the promotion of autophagy and resistance in gastric cancer cells (Additional file 1: Figure S1; Additional file 2: Figure S2).

## Discussion

To date, chemotherapy resistance remains one of the major obstacles in cancer treatment [24]. Our group has long been committed to the treatment and research of gastric cancer. In previous studies, TSPAN9 was found to be involved in tumor invasion and metastasis [16]. Immunohistochemistry and Western blotting have shown that low levels of TSPAN9 expression were detected in clinical tumor specimens and gastric cancer cell lines, but elevated TSPAN9 expression was associated with poor prognosis, which encouraged us to conduct more in-depth research on TSPAN9. There are few studies on TSPAN9 in cancer, and existing research has focused on its role in inflammation [14, 25]. Tetraspanins are known to bind to integrins, receptor tyrosine kinases (RTKs) and intracellular signaling molecules and transduce signals through cancer-associated signaling molecules, thereby affecting cell adhesion, migration and invasion [26–28]. According to the literature, 5-FU-resistant cells have high levels of TSPAN9 compared to their parental cells, which provides a suitable model for analyzing the mechanism. In the present study, knocking down TSPAN9 expression in drug-resistant cells and increasing TSPAN9 expression in parental cells indicated that TSPAN9 plays a key role in 5-FU resistance. At the same time, we found that high levels of TSPAN9 contribute to increased autophagy in gastric cancer cells. Many studies have shown that the high levels of autophagy in tumor cells may be one of the causes of chemotherapy resistance [29–31]. Therefore, we inhibited autophagy using the autophagy inhibitor 3-MA and observed inhibition of the protective effect of TSPAN9 on gastric cancer cells. These findings show a novel mechanism by which TSPAN9 protects against the cytotoxic effects of 5-FU through increasing the level of autophagy in gastric cancer cells. The PI3K–Akt–mTOR pathway is a classical inhibitory pathway of autophagy that plays a significant role in the development and reversal of chemoresistance [32, 33]. Our results indicate that TSPAN9 promotes autophagy and enhances the resistance of gastric cancer cells to 5-FU by inhibiting the activation of PI3K–Akt–mTOR signaling. To analyze how TSPAN9 regulates the autophagy pathway, immunoprecipitation assays were performed, which confirmed that TSPAN9 interacts with the p55 subunit of PI3K to inhibit PI3K catalytic activity. The phosphorylation of TSPAN family members is dependent on PI3K binding, so we further investigated whether the phosphorylation status of TSPAN9 is the key to its binding to PI3K. We used KinasePhos software to analyze the possible phosphorylation sites of TSPAN9, and the results indicated that Tyr153 is the site that is most likely mutated to prevent phosphorylation. By constructing a Tyr153 mutant plasmid and transfecting it into gastric cancer cells, we confirmed that phosphorylation of Tyr153 is required for binding PI3K.

In conclusion, this study showed that TSPAN9 and PI3K bind to each other in gastric cancer cells, which inhibits the activity of the PI3K–Akt–mTOR pathway and consequently enhances the level of autophagy and promotes 5-FU resistance. Phosphorylation of TSPAN9 at Tyr153 is key to promoting the binding of TSPAN9 to PI3K. Through this mechanism, TSPAN9 protects gastric cancer cells from 5-FU-induced cell death and increases drug resistance. This work provides an important contribution to our understanding of the mechanism by which TSPAN9 enhances gastric cancer cell resistance to chemotherapy.

## Conclusions

We have demonstrated that TSPAN9 induces 5-FU resistance by increasing autophagy in gastric cancer. In addition, phosphorylation of TSPAN9 promotes its binding to PI3K. Therefore, we have highlighted that TSPAN9 and other tetraspanins may represent novel therapeutic targets for the treatment of gastric cancer.

## Supplementary information

**Additional file 1: Figure S1.** AGS, AGS/R, MGC803 and MGC803/R cells were subjected to transmission electron microscopy to detect autophagosome.

**Additional file 2: Figure S2.** Densitometric analysis normalized to ACTB demonstrating the LC3-II levels in AGS, AGS/R, MGC803 and MGC803 cells and the effect of TSPAN9. Values are means ± SEM of 4 to 6 experiments. *p < 0.05, **p < 0.01, compared to control or vehicle.

## Data Availability

Not applicable.

## Abbreviations

TM4SF: transmembrane 4 superfamily
FU: 5-fluorouracil
LEL: large extracellular loop
SDS: sodium dodecyl sulfate
PAGE: SDS-polyacrylamide gel electrophoresis
PBS: phosphate-buffered saline
OsO4: osmium tetroxide
TEM: electron microscopy
MOI: multiplicity of infection
MA: 3-methyl adenine
PTMs: posttranslational modifications
PPIs: protein–protein interactions
TKI: tyrosine kinase inhibitor
