# Supplementary figures and images for: TSPAN9 suppresses the chemosensitivity of gastric cancer to 5-fluorouracil by promoting autophagy
Source: Cancer Cell Int. 2020 Jan 3;20:4. doi: 10.1186/s12935-019-1089-2 (PMC6942356; doi:10.1186/s12935-019-1089-2)

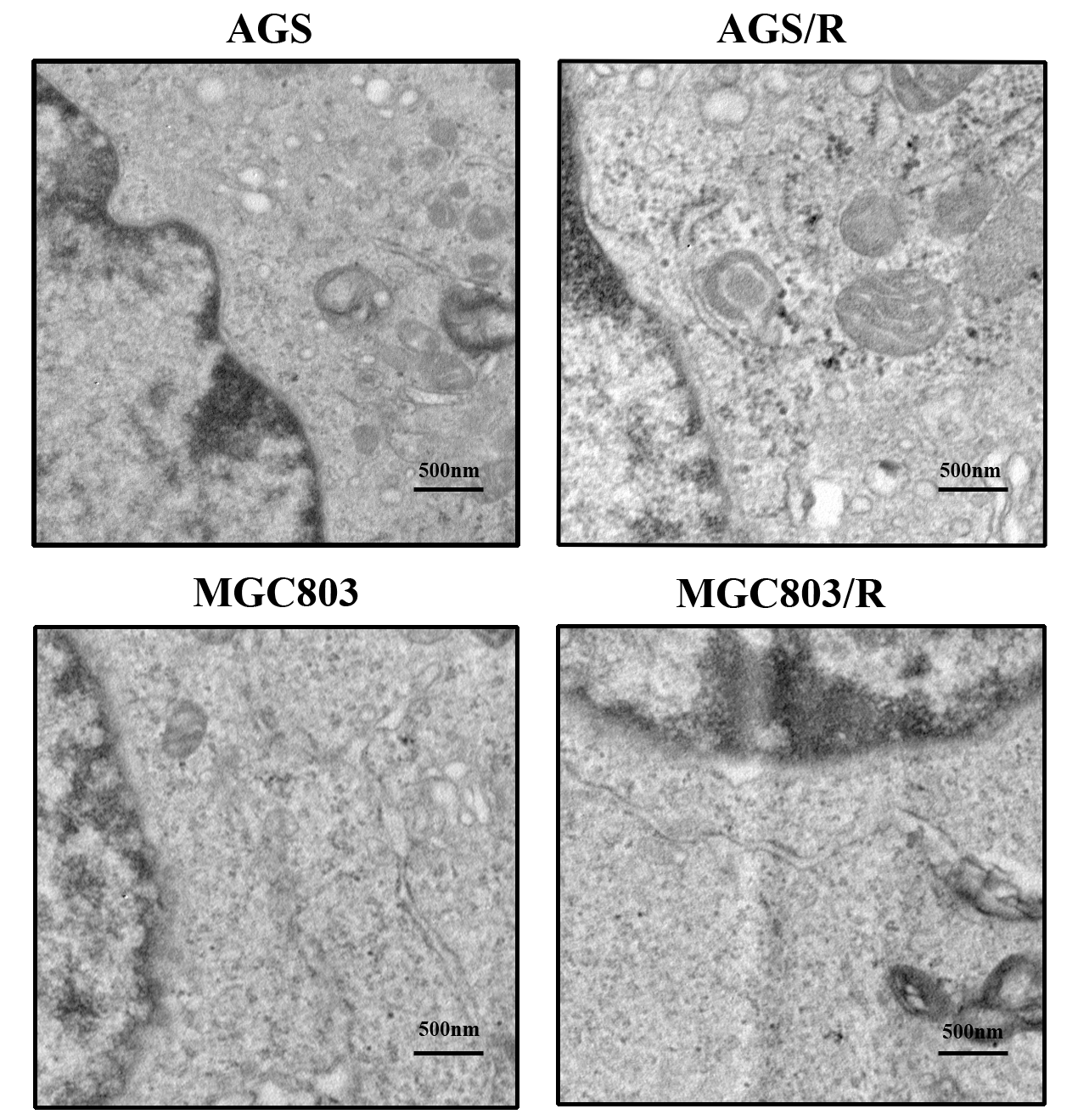

Supplement: Supplementary file 1 — Additional file 1: Figure S1. AGS, AGS/R, MGC803 and MGC803/R cells were subjected to transmission electron microscopy to detect autophagosome. [file 12935_2019_1089_MOESM1_ESM.tif]

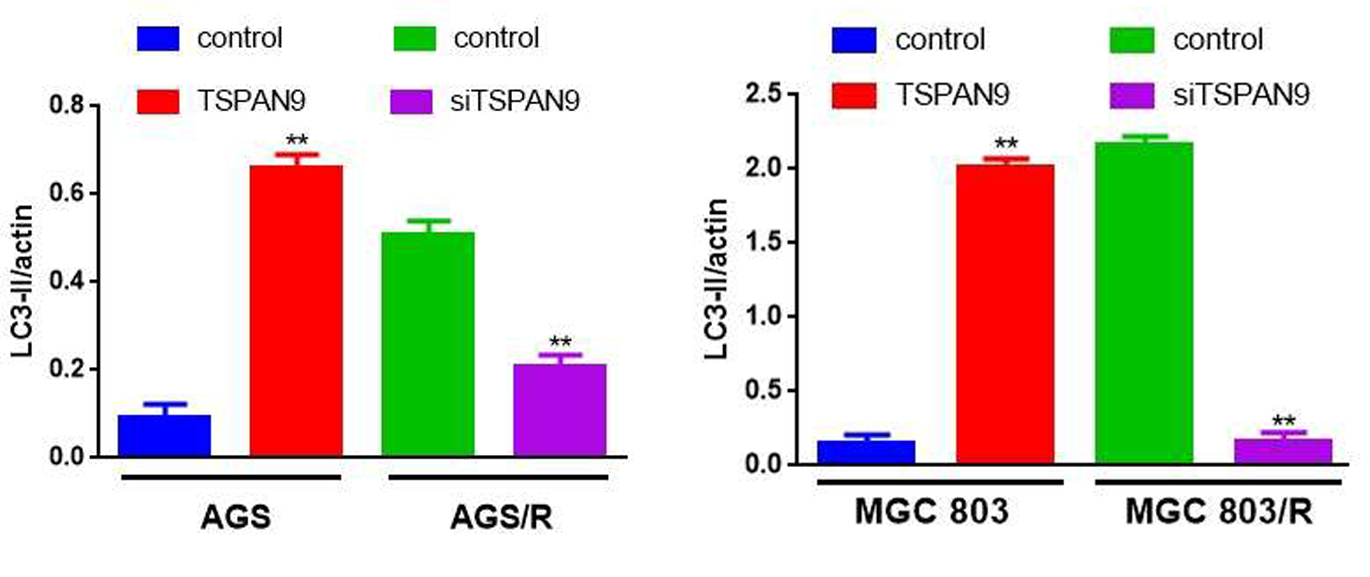

Supplement: Supplementary file 2 — Additional file 2: Figure S2. Densitometric analysis normalized to ACTB demonstrating the LC3-II levels in AGS, AGS/R, MGC803 and MGC803 cells and the effect of TSPAN9. Values are means ± SEM of 4 to 6 experiments. *p < 0.05, **p < 0.01, compared to control or vehicle. [file 12935_2019_1089_MOESM2_ESM.tif]
